# Life-history evolution in the anthropocene: effects of increasing nutrients on traits and trade-offs

**DOI:** 10.1111/eva.12272

**Published:** 2015-06-13

**Authors:** Emilie Snell-Rood, Rickey Cothran, Anne Espeset, Punidan Jeyasingh, Sarah Hobbie, Nathan I Morehouse

**Affiliations:** 1 Department of Ecology, Evolution and Behavior, University of MinnesotaTwin Cities, MN, USA; 2 Department of Biological Sciences, University of PittsburghPittsburgh, PA, USA; 3 Department of Biological Sciences, Southwestern Oklahoma State UniversityWeatherford, OK, USA; 4 Department of Biology, University of NevadaReno, NV, USA; 5 Department of Zoology, Oklahoma State UniversityStillwater, OK, USA

**Keywords:** life-history traits, nitrogen, phosphorus, signals, trade-offs

## Abstract

Variation in life-history traits can have major impacts on the ecological and evolutionary responses of populations to environmental change. Life-history variation often results from trade-offs that arise because individuals have a limited pool of resources to allocate among traits. However, human activities are increasing the availability of many once-limited resources, such as nitrogen and phosphorus, with potentially major implications for the expression and evolution of life-history trade-offs. In this review, we synthesize contemporary life history and sexual selection literature with current research on ecosystem nutrient cycling to highlight novel opportunities presented by anthropogenic environmental change for investigating life-history trait development and evolution. Specifically, we review four areas where nutrition plays a pivotal role in life-history evolution and explore possible implications in the face of rapid, human-induced change in nutrient availability. For example, increases in the availability of nutrients may relax historical life-history trade-offs and reduce the honesty of signaling systems. We argue that ecosystems experiencing anthropogenic nutrient inputs present a powerful yet underexplored arena for testing novel and longstanding questions in organismal life-history evolution.

## Introduction

For decades, life-history theory has focused on why organisms vary in major fitness-related traits such as offspring number, age at first reproduction, body size, and life span (Stearns 1992; Roff 2001). Such life-history variation is underlain in part by trade-offs that arise because individuals have a limited pool of resources to allocate among traits (Zera and Harshman 2001; Flatt and Heyland 2011). However, human activity is increasing the availability of many nutrients that were previously limiting resources in ecosystems (Vitousek et al. 1997a,b; Smith 2003), which has major implications for understanding the expression and evolution of life-history trade-offs in human-impacted landscapes. In this review, we first summarize ecological studies that highlight how humans are changing the availability of key nutrients. Next, we review four areas where changing nutrient availability is likely to affect life-history evolution: plasticity in life-history traits, the physiological basis of life-history trade-offs, the honesty of sexual traits, and genotype-by-environment interactions in life-history traits. For each of these areas, we review some of the relevant literature before discussing the implications for understanding life-history traits and evolution in the face of rapid and human-induced changes in nutrient availability. Overall, we argue that anthropogenic changes in nutrient availability present unique and powerful opportunities to test fundamental questions about life-history evolution (Table1: H1–6). For instance, anthropogenic nutrient inputs may help to address the longstanding question of why life-history traits and trade-offs vary within and between species. At the same time, understanding evolutionary responses of life histories to changing nutrients should enable better predictions of how populations will respond to rapid environmental change (Table1: H12–16).

**Table 1:** Summary of questions and hypotheses about life-history evolution presented by anthropogenic nutrient change

Questions about life-history traits and strategies
Why do species or populations vary in life-history traits?
H1: Anthropogenic changes in nutrients may allow some species or populations to allocate more to *all* life-history traits (e.g. offspring number & quality, brain size)
Why do life-history trade-offs vary in intensity?
H2: Human-caused increases in nutrients over time and space may obscure trade-offs (while decreases in nutrients may make trade-offs more pronounced)
H3: Anthropogenic increases in one nutrient may result in a novel limiting nutrient that reveals new trade-offs and/or genetic variation
Why not invest maximally in a trait closely tied to fitness?
H4: Anthropogenic change in one nutrient may result in a novel nutrient limiting trait expression
H5: In high nutrient environments, relaxed selection on nutrient acquisition and/or assimilation may lead to lessened trait expression when anthropogenic nutrient increases are lessened
Why do species or populations vary in life-history *plasticity*?
H6: For some nutrients, anthropogenic change will increase the spatial and temporal variability of nutrient availability, selecting for greater plasticity in response to that nutrient
Questions about sexual traits
Why does investment in sexual traits vary within and between species?
H7: Female choosiness may increase with nutrient status due to increased resources dedicated to choice and/or increased self-assessment of reproductive value by females
H8: Increasing nutrients may lead to increased population sizes/densities, which in turn can result in increased selection on traits involved in male-male competition
How do complex, honest signals evolve as part of the overall life-history strategy of an organism?
H9: Honesty of nutrient-limited sexual traits should decline with anthropogenic nutrient increases, potentially leading to relaxed selection on sexually selected traits and possibly individual quality
H10: As nutrient availability changes, selection shifts to favor novel signals linked to new resource limitations (signal diversification)
H11: As nutrient availability changes, selection favors increasing allocation to an existing signal (signal elaboration)
Questions about responses to rapid environmental change
Why might populations and species show different responses to rapid and novel anthropogenic environments?
H12: Increases in nutrient availability may allow some populations to allocate more to life-history traits that affect both survival (e.g. plasticity) and evolution (e.g. fecundity) in novel environments
H13: Changes in allele frequencies within populations may be driven by spatio-temporal nutrient variation that affects life-history traits independent of any other factors that vary across individuals
H14: Standing genetic variation in life-history responses to nutrition (G × E) may contribute to rapid evolutionary changes in nutrient acquisition, assimilation and allocation in novel nutrient environments
H15: Evolutionary processes such as population divergence may be sped up by anthropogenic increases in nutrients; alternatively, nutrient change may reduce divergence by introducing fluctuating selective regimes
H16: Ecological changes in community structure due to outcomes of competition in high nutrient environments may bias which species survive and diversify in high nutrient environments

## The Ecology of Anthropogenic Nutrient Change

**Figure 1:**
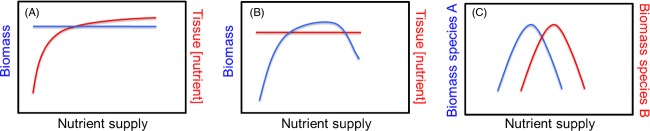
Possible effects of changes in nutrient availability on nutrition at higher trophic levels. Nitrogen, phosphorus, calcium, and sodium from human activities are readily available to organisms. Changes in nutrient availability can affect both the nutritional quality and quantity of resources, because of individual-level responses to altered nutrient supply (e.g. increased growth or tissue nutrient concentrations) and changes in species composition (e.g. arising from variation in competitive ability for nutrients in resources). It is assumed that every species is adapted to some level of nutrients and performs poorly when nutrient levels are either so low they are limiting or so high they are toxic. As nutrient levels shift, competitive interactions among species result in altered community dynamics (Bobbink et al. 2010). These changes result in three possible changes in nutrition that can occur as a result of shifts in nutrient availability: (A) Altered resource quality because of changes in the amount of nutrients per individual, such as changes in leaf nutrient concentrations (moving up the red curve); (B) Altered resource quantity results in more nutrients per unit area, such as changes in biomass without accompanying changes in individual-level nutrients (moving up the blue curve prior to when nutrient levels increase to a stressful level); (C) Altered resource quality because of changes in community composition, such as change in the dominance of a higher quality resource (species B is of higher quality than A). Of course, in many cases, both the quality and quantity of nutrition can change.

Humans are altering the way nutrients enter and are cycled in ecosystems (briefly summarized here, but reviewed extensively by Vitousek et al. 1997a,b; Smil 2000; Galloway et al. 2004, 2008). Changes in nutrient availability can affect the nutritional quality or quantity of resources either directly or through interactions with other species (Fig.1). This review focuses on increases in the quality or quantity of resources for a given species or set of species (i.e. prior to the point of nutrient stress). While organismal nutrition is often addressed in relation to the macromolecular composition of food items (e.g. proteins, carbohydrates, Simpson and Raubenheimer 2012), we have chosen to discuss nutrition from an elemental or stoichiometric perspective (*sensu* Sterner and Elser 2002) because it is at this level that we best understand how humans are affecting nutrient cycling in ecosystems. In particular, we focus on nitrogen and phosphorus as case studies of macronutrients and sodium and calcium as case studies of micronutrients; however, humans are affecting the availability of a wide range of nutrients and other resources, including carbon, lipids, and water. Other nutritionally explicit approaches that consider resources at a macro level (e.g. proteins versus nitrogen), such as the geometric framework for nutrition (Simpson and Raubenheimer 2012), may offer additional advantages and complementary data in empirical work.

### Macronutrients: nitrogen and phosphorus

Human activities are affecting both nitrogen and phosphorus inputs to ecosystems; this is of ecological and evolutionary interest because nitrogen and phosphorus are the nutrients that most commonly limit primary producers worldwide (Elser et al. 2000). In addition, nitrogen and phosphorus effects may propagate through food webs, altering the productivity, abundance, composition, and chemistry of higher trophic levels, although nutrient-induced changes in species composition toward more unpalatable or defended species may prevent enhanced productivity at any given trophic level from propagating to higher levels (Peterson et al. 1993; Deegan et al. 2002; Borer et al. 2006; Cross et al. 2007; Davis et al. 2010; Finlay 2011).

Fossil fuel combustion, fertilizer production and use, cultivation of legume crops, and deforestation create and mobilize reactive nitrogen, oxidized and reduced nitrogen forms that are largely biologically available (Galloway et al. 2004). Many of these forms of nitrogen move readily through the atmosphere, ground and surface water, and additionally through the export of food, livestock feed, and fertilizer (Galloway et al. 2008). Thus, regions with heavy industry, agriculture, or consumption of food (by livestock or people) are associated with elevated downwind atmospheric nitrogen deposition and downstream riverine nitrogen. Nitrogen effects can be not only local, but also far-reaching due to long distance transport: forms of nitrogen can move down rivers from agricultural or industrial areas to coastal zones or can be transported in foods from areas of production to areas of consumption (Elser et al. 2009).

Human activities are also altering phosphorus cycling and elevating phosphorus inputs to ecosystems, particularly aquatic ecosystems. Phosphorus is much less mobile than nitrogen, as it lacks a measurable gaseous phase and does not leach readily through soils. Unlike nitrogen, phosphorus is transported primarily to waterways in erosion and runoff and through the atmosphere as dust (and only in small amounts). Humans have altered the global cycling of phosphorus by promoting erosion and runoff through land clearing, cultivation, and overgrazing; applying inorganic phosphorus fertilizer; producing phosphorus-containing detergents that end up in wastewater; and increasing (and geographically concentrating) the consumption of food, feed, and associated phosphorus-rich waste by humans and livestock (Smil 2000). Elevated aquatic inputs of both phosphorus and nitrogen come from both nonpoint sources (associated mainly with agriculture) and point sources, such as wastewater treatment plants. However, large parts of the world are not connected to sanitary sewers and may have high nitrogen and phosphorus inputs to waterways in the form of raw sewage. Even where sewage is treated (Carpenter et al. 2011), wastewater treatment varies greatly in nitrogen and phosphorus removal, so effluent may still contain high concentrations of either nitrogen or phosphorus (Carey and Migliaccio 2009).

### Micronutrients: calcium and sodium

Along with macronutrients, humans are altering the availability of many micronutrients, such as sodium and calcium. Sodium is a major limiting micronutrient for many animals, and salt requirements drive behavior in both vertebrates and invertebrates (Smedley and Eisner 1995; Kaspari et al. 2008). Humans are increasing sodium availability locally through road salt runoff and agricultural practices (Findlay and Kelly 2011). While much of the existing road salt literature focuses on the toxic effects of increasing chloride levels due to road salt application (Kaushal et al. 2005), increased sodium availability may have entirely different effects for some organisms (Jackson and Jobbagy 2005). As a cation, sodium in road runoff is more likely than chloride to be retained in soils (which are negatively charged in regions where road salt is applied; Ramakrishna and Viraraghavan 2005). Increased sodium availability in areas near paved roadways (Kelting et al. 2012) has already been shown to alter foraging ecology as animals attempt to quench their salt needs (Laurian et al. 2008; Kaspari et al. 2010) along with the development of sodium-limited traits in roadside-feeding herbivores (Snell-Rood et al. 2014). The broader effects of changing sodium availability on nutritional ecology and life-history evolution remain to be studied. Historically, sodium has been most limited at high elevations and areas far from the ocean (Dudley et al. 2012), but this is changing due to human activity.

Calcium is another limiting micronutrient for many organisms, from plants to vertebrates (Perrins 1996; White and Broadley 2003). Variation in calcium availability has major impacts on the ecology of species with high calcium requirements including certain worm species (Reich et al. 2005), snails (Skeldon et al. 2007), and birds (Wilkin et al. 2009). To date, much work has focused on how human-induced changes in soil acidity have decreased available calcium and negatively impacted species (Juice et al. 2006). However, in some cases, humans have increased available calcium. Anthropogenic calcium inputs arise from cement use, for instance, leaching into urban waterways from cement pipes (Wright et al. 2011) or atmospheric fallout associated with cement plants, limestone quarrying, or building destruction (Branquinho et al. 2008). This results in local increases in calcium availability; for instance, calcium levels are elevated in urban areas (Pouyat et al. 2008). Whether this anthropogenic increase in calcium availability has effects on life-history trade-offs remains unknown, but explorations on this front will need to consider correlated changes in toxins such as heavy metals (Marcotullio 2011).

## Life-History Traits in the Face of Anthropogenic Changes in Nutrients

**Figure 2:**
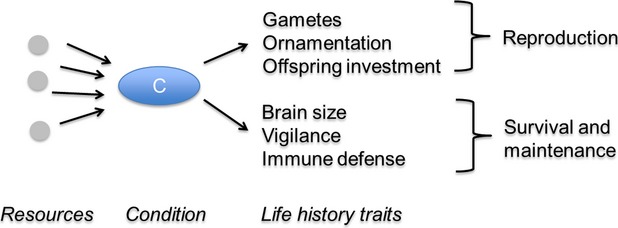
Operational definition of life-history traits. In this review, we adopt a broad definition of life-history traits as any trait tied to reproduction, survival or somatic maintenance. In the past, life-history traits have been traditionally defined as traits that compose the life table of a population—traits tied closely to fitness such as growth, body size, annual reproductive rates, and survival. In more recent decades, other traits closely tied to fitness have been argued to fall under the general umbrella of life-history traits. In particular, sexually selected traits can be thought of as an investment in reproductive effort and thus understanding their expression requires a life-history approach (Andersson 1994; Badyaev and Qvarnström 2002). In fact, the intimate relationship between sexually selected traits and life history was realized early in evolutionary ecology as an explanation for why the sexes differ in life-history traits (Orians 1969). Sexually selected traits are often expensive to build and maintain and may compete strongly with other life-history traits for resources (Ryan 1988; Balmford et al. 1993). This competition for shared resources can ultimately lead to trade-offs between investment in sexually selected traits and other life-history traits (Gustafsson et al. 1995; Kotiaho 2000). Therefore, these two types of traits are likely to be linked and both are expected to be sensitive to fluctuations in the resource environment. Similar arguments could be made for other traits closely tied to fitness such as brain size. In particular, the cognitive buffer hypothesis links brain size to survival in the face of environmental variation and complex decision making (Kaplan and Robson 2002; Sol 2009; Møller and Erritzoe 2014). This, combined with observations of trade-offs between brain size and other traits such as gut length or muscle mass (Isler and van Schaik 2006; Kotrschal et al. 2013), suggest that brains should also be considered a life-history trait. For the purposes of this review, life-history allocation refers to how an individual or genotype allocates limited resources to the entire set of life-history traits that determine their overall fitness.

Changes in nutrient availability have major implications for the development and evolution of life-history traits, and trade-offs between these traits. Here, we broadly define an organism’s life history as those traits that directly affect an individual’s survival and production of offspring (see Fig.2, Reznick et al. 2000) including classic traits such as fecundity and life span, and, in some cases, additional traits such as brain size and ornamentation (Badyaev and Qvarnström 2002; Sol 2009). Resources and nutrients play a major role in many aspects of life-history theory. Here, we focus on four of the best-studied, but interrelated, concepts in life-history evolution where resource and nutrient availability play an important role: plasticity in life-history traits, life-history trade-offs, honesty of signals, and genotype-by-environment interactions. Each of these contexts has consequences for understanding life-history traits and life-history evolution in the anthropocene. Understanding how increasing nutrient availability affects the expression of life-history traits and trade-offs has implications for predicting the ecological and evolutionary responses of populations in the anthropocene.

### Life-history plasticity in response to nutrition

**Figure 3:**
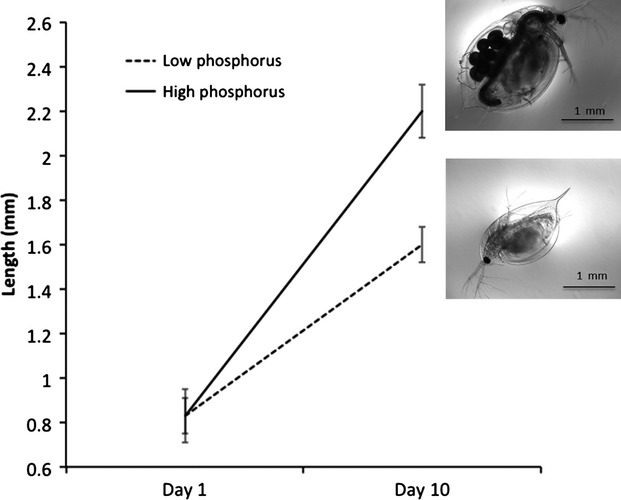
The influence of nutrition on life-history traits—*Daphnia* as an example. It seems almost a given that nutrient availability should affect life-history traits such as offspring number and life span, here illustrated by *Daphnia*, which feed on algae. Algal phosphorus (P) content often closely tracks inorganic P supply (Rhee 1973), although algal cells continue photosynthesis and become carbon (C) rich (Tillberg and Rowley 1989). Consequently, *Daphnia* inhabiting lakes with varying P supply experience contrasting diets in terms of both P and C (Sterner and Hessen 1994). Such variation has major consequences on key life-history traits of *Daphnia*. Specifically, compared to daphniids feeding on high P algae, those in low P grow slower, delay reproduction, reproduce at a smaller size, and produce smaller broods (e.g. Lurling and Van Donk 1997). Importantly, such life-history shifts are not only driven by P availability, but also due to excess C (Anderson et al. 2005). This figure shows results (mean ± SD) from Jeyasingh and Weider (2005) where <12 h-old clonal sisters of *D. pulex* were exposed to contrasting P supply conditions. The growth and fecundity penalties of low P after 10 days are quite apparent. Note that total amount of energy in both dietary treatments were the same (1 mg C L^−1^ day^−1^). While variable nutrition clearly affects the expression of life-history traits in *Daphnia*, the effects of nutrition may vary with the specific nutrients considered, sex- and developmental stage-specific responses to changes in nutrients, and differences across genotypes in nutrient acquisition ability. Furthermore, nutrient variation may differentially influence generalists versus specialists and active foragers versus passive feeders.

Nutrition has innumerable effects on the development of life-history traits (Nylin and Gotthard 1998). Many studies have demonstrated the sensitivity of life-history traits, such as growth rate and fecundity, to changes in resource quantity and quality (Martin 1987; Vanni and Lampert 1992; Boggs and Ross 1993; Twombly et al. 1998; see Fig.3 for *Daphnia* as an example). For instance, fertilizer application affects the growth, fecundity, and survival of not only plants, but also their herbivores (Hauch 1984). Several studies have identified specific diet components that are important for life span and fecundity, such as protein and carbohydrates (Lee et al. 2008), the effects of which interact with sex (Maklakov et al. 2008). Growth, fecundity, and other life-history traits are often sensitive to phosphorus or nitrogen availability (Sterner 1993; Elser et al. 2001; Jeyasingh and Weider 2005; Morehouse and Rutowski 2010a). While performance generally improves on diets enriched with phosphorus and nitrogen, this is not always the case, especially if the diet becomes stoichiometrically unbalanced (Boersma and Elser 2006). Collectively, these studies highlight the fact that life-history traits are often extremely sensitive to nutritional perturbations.

**Figure 4:**
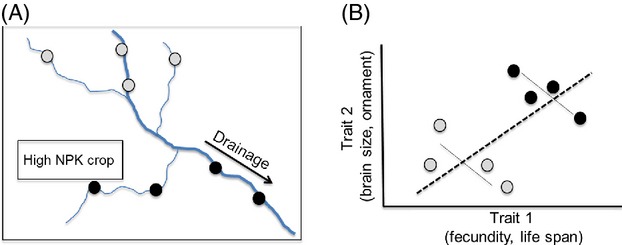
How anthropogenic nutrient increase may obscure underlying trade-offs. (A) In this landscape, runoff from a field with high fertilizer application (nitrogen, phosphorus, and potassium inputs) enters the watershed. Imagine genotypes of an aquatic species (circles) sampled across this region: those downstream of the source (in black) may be heavily affected, while those upstream of the source may be less affected (light gray). (B) Variation in nutrient inputs across this landscape may result in a positive correlation between life-history traits (dotted line) because some genotypes have greater overall nutrition (black), even if there is an underlying trade-off within each resource level (solid lines).

Nutrient-induced changes in life-history traits (Table1: H1) have consequences for how organisms compete with each other and resulting community dynamics (Sterner and Elser 2002; Tylianakis et al. 2008). These environmental effects also have evolutionary implications. Given that anthropogenic increases in nutrient availability are often spatially heterogeneous, it is possible that some individuals may become more fecund or longer lived solely as a consequence of higher local resource availability rather than the breeding value of their genotype (Fig.4; Table1: H13). Spatial heterogeneity of resource richness may lead to spatial sorting of phenotypes, genotypes, and/or species interactions that should have important implications for the selective landscape that metapopulations experience (e.g. Leibold et al. 2004; Shine et al. 2011). Thus, resource-mediated changes in life-history traits and population dynamics could create novel and spatially complex selection dynamics which are only just starting to be investigated (e.g. wild sheep, Wilson et al. 2006). Alternatively, more persistent or spatially homogeneous nutrient inputs into ecosystems may result in changes to historically important selective pressures through, for example, relaxed selection on traits linked to previously limiting resources (Snell-Rood et al. 2010; Table1: H5). However, such weakened selection may lead to increased emphasis on other newly limiting resource pools (Table1: H4). The ensuing directional selection may reshape trophic dynamics, leading to adaptive modifications of a focal organism’s foraging strategies or resource allocation rules (see below).

### Nutrition as a mediator of life-history trade-offs

**Figure 5:**
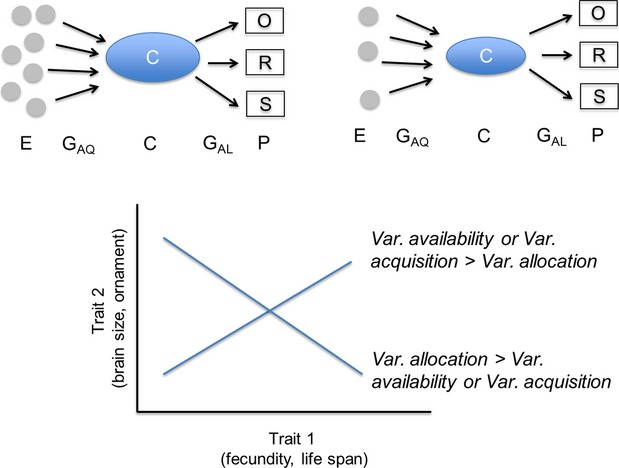
Routes by which nutrition may affect life-history trade-offs. Variation in life-history traits (phenotypes, ‘P’) can come through variation in resource availability (the environment, ‘E’), genetic variation in resource acquisition (‘G_AQ_’), which both influence an individual’s condition (‘C’) or genetic variation in how resources are allocated across life-history traits (‘G_AL_’). Variation in resource availability or acquisition can generate positive life-history trait correlations across genotypes even when there are underlying trade-offs across traits—in other words, higher condition individuals can simply allocate more resources to all traits such as survival (‘S’), reproduction (‘R’), or ornamentation (‘O’). When variation in acquisition is higher than variation in allocation, position relationships will be seen; when variation in acquisition is less than variance in allocation, negative relationships will be seen. Figure modified from (Rowe and Houle 1996; Morehouse 2014).

Natural selection shapes how an organism allocates resources among life-history traits (‘principle of allocation’, Cody 1966; Levins 1968). Variation in life-history traits is the result of both the trade-offs that occur due to the competing demands of traits and the ecological context that determines which trait combinations are favored by natural selection (van Noordwijk and de Jong 1986; Reznick et al. 1990). These trade-offs are often the result of traits competing for internal limiting resources (Zera and Harshman 2001). The ‘y-model’ of life-history theory posits that resources allocated to one life-history trait are not available for allocation to other life-history traits, resulting in trade-offs across individuals or genotypes (see Fig.5). However, measuring such trade-offs depends on resource availability—if individuals have greater access to a particular nutrient or are better able to acquire that nutrient, variation between individuals in overall nutrition can obscure any underlying trade-off between traits (van Noordwijk and de Jong 1986; Reznick et al. 1990; de Jong and van Noordwijk 1992). Thus, trade-offs across life-history traits are often apparent only after accounting for variation in nutrient acquisition across genotypes and environments (Mole and Zera 1994; Glazier 1999; Reznick et al. 2000; Sgrò and Hoffmann 2004; King et al. 2011a,b).

Underlying trade-offs may be obscured by anthropogenic changes in once-limited nutrients that create variation across individuals in access to resources (Fig.4; Table1: H2). Increases in available nutrients may make trade-offs less pronounced assuming that an increase in nutrient availability decreases variation across individuals in resource acquisition. In some cases, increases in nutrient availability may interact with foraging behavior such that variation across individuals in nutrient acquisition is maintained, in which case the intensity of trade-offs might not vary. For some nutrients, there may also be an increase in the *variability* of nutrient availability over space and time, leading to positive correlations between competing traits when observed at the level of populations (Fig.4).

Anthropogenic nutrient change may affect the evolutionary trajectory of populations through its relaxation of life-history trade-offs (Table1: H12). Given the rate of rapid environmental change occurring today, there has been much discussion about how populations will respond to climate change, habitat conversion, and other human-induced changes to the environment (Parmesan 2006; Bell and Collins 2008). Some species may be able to rapidly respond to environmental change. For example, species capable of rapid population growth tend to show pronounced evolutionary responses to rapid environmental change (Reznick and Ghalambor 2001). Similarly, species that exhibit high developmental plasticity in traits related to coping with environmental changes, such as learning ability, can flexibly adjust their behavior and development to novel conditions (Sol et al. 2005). Trade-offs between reproduction and learning ability (Snell-Rood et al. 2011; Kotrschal et al. 2013) suggest that, in some cases, a population’s evolutionary response may be at odds with its developmental response to a new environment. However, if life-history trade-offs are relaxed under high nutrient conditions, the severity of these trade-offs may be reduced in some modern environments, in the process liberating specific populations to express both developmental and evolutionary responses that are beneficial under the novel conditions. Alternatively, limited availability of other nutrients may still enforce or lead to new trade-offs (Table1: H3), leading to a mosaic of rapid adaptive responses and constraints predicated on shifting resource dynamics. Thus, attention to anthropogenic effects on multiple nutrient currencies will provide critical opportunities to understand and potentially predict the evolutionary trajectories of focal populations.

### Nutrition and the honesty of sexual signals

An area of life-history thinking that has been highly developed with respect to nutrition is that of the condition dependence of sexual signals (Morehouse 2014). The utility of traits as indicators of individual quality often relies on strong condition dependence of traits (Zahavi 1975; Iwasa and Pomiankowski 1991; Andersson 1994). Condition-dependent traits are thought to inform prospective mates or competitors of an individual’s ability to acquire, assimilate, and/or spend key resources, processes that should be linked to fitness. Because such traits are likely to be most informative when they signal resources in high demand and short supply, the expectation is that sexual selection should favor indicator traits linked to limiting resources. For example, this argument forms the basis for favored explanations of the pervasive use of diet-derived carotenoids in colorful ornaments of birds and fish (Hill and Montgomerie 1994; Grether et al. 1999; Velando et al. 2006; Svensson et al. 2006). Similar arguments have been made for other resource pools such as nitrogen (Morehouse et al. 2010; Morehouse and Rutowski 2010b), phosphorous (Bertram et al. 2006, 2009; Cothran et al. 2012), brightly colored objects used to decorate bowerbird bowers (Borgia et al. 1987), and energy available for condition-dependent motor displays (Byers et al. 2010) such as claw waving in fiddler crabs (Jennions and Backwell 1998). In addition to ornaments, sexually selected armaments used in pre- or postcopulatory male–male competition are often similarly condition dependent. Weaponry used in male–male combat, such as ungulate antlers or beetle horns, often draw heavily on resource pools that can be limiting in diets (Moczek and Nijhout 2004). For example, the extreme mineral requirements of ungulate antlers can lead to temporary osteoporosis as males ‘borrow’ calcium from skeletal pools during periods of antler growth (Baxter et al. 1999).

We might expect that as once-limited resources increase in availability, the honesty of signals based on these resources will decrease (Table1: H9), assuming that an increase in nutrient availability corresponds to a decrease in variability across individuals in resource acquisition. Indeed, some experiments suggest that ornament attractiveness is an indicator of male quality only under low nutrient conditions (David et al. 2000; Tolle and Wagner 2011). The potential impact of increased supply of once-limiting resources on sexually selected traits depends on the timescale considered. On short timescales (several generations), we might expect that individuals in resource-rich environments should all be able to produce highly exaggerated ornaments. Thus, in the short term, sexually selected traits may be powerful indicators of nutrient pollution (Hill 1995). Such nutrient pollution may lead to transient relaxation of sexual selection on ornamented males by removing the ability of females to detect differences in male genetic quality based on ornament expression (Akre and Johnsen 2014) similar to that observed in fish as humans have altered water turbidity (Seehausen et al. 1997; Engstrom-Ost and Candolin 2007).

If anthropogenic nutrient inputs persist for longer periods, the evolutionary consequences for sexual selection are likely to be more complex. For instance, if genetic variation in individual quality and ornament expression is still visible despite an increase in resource availability, and individuals of the choosy sex employ an open-ended preference function, this may result in rapid escalation of ornament exaggeration (Table1: H11), a liberation of the Fisherian runaway model from resource limitation. However, if individuals in resource-rich populations produce high but indistinguishable levels of ornament expression, this should lead to the disappearance of preferences based on the ornaments whose utility has been lost in the context of nutrient pollution. For instance, agricultural populations of cabbage white butterflies, which have seen relatively greater increases in nitrogen with fertilizer application, allocate more to nitrogen-based wing pigments used in mate choice; however, it is unclear to what extent this compromises signal honesty (A. Espeset, C. Roy, and E. C. Snell-Rood, in revision). Persistent increases in the availability of once-limited nutrients may decrease the utility of ornaments as indicators, resulting in sexual selection favoring either novel or preexisting traits indicative of other more pertinent resource pools. This shifting selection may result in ornament ‘proliferation’, where ornaments become more complex or multimodal over evolutionary time (Table1: H10; Badyaev 2004; Hebets and Papaj 2005).

Changing nutrient availability should affect not only the honesty of traits of the signaler, but also the likelihood that a receiver will discriminate among possible mates or competitors. For instance, resource state can influence the likelihood that a female will engage in mate choice as well as the preference function she applies to this task (Jennions and Petrie 1997; Widemo and Sæther 1999; Hunt et al. 2005; Cotton et al. 2006). Increases in the supply of key resources may increase female fecundity, perceived reproductive value, and likelihood that she will employ strong preference functions when evaluating potential mates (Table1: H7). This should in turn lead to stronger directional selection on male ornamentation, potentially reinforcing the volatility, and strength of sexual selection in populations experiencing novel resource inputs. In some cases, variation in resource availability may even result in role reversals between sexes shifting which sex is relatively choosier (Gwynne and Simmons 1990).

Finally, anthropogenic nutrient change may also have implications for condition-dependent weaponry used in male–male combat. Population increases in the size of armaments as a result of increasing nutrient availability may lead to increases in the frequency of male–male combat resulting from increased uncertainty over male dominance. Alternatively, resource-driven increases in effective population sizes may simply increase the opportunity for, and therefore strength of, competition among individuals for access to mates, leading to stronger selection for traits related to intrasexual competition (e.g. in beetles, Moczek 2003; Table1: H8).

### G × E in life-history traits and complex responses to nutrient change

In many cases, investment in life-history traits depends not only on the nutritional environment, but also on interactions between genotype and nutritional environment. Genotype-by-environment interactions suggest there is often ample genetic variation in life-history responses to nutrition (Stratton 1994; Tomkins 1999; Hairston et al. 2001; Bergland et al. 2008). Such patterns may arise out of genetic variation in nutrient assimilation efficiency or variation in allocation strategies in different nutritional environments (Baligar and Duncan 1990; Evans 1993; Morehouse 2014). Extreme changes in nutrition may reveal once cryptic genotype-by-environment interactions [‘hidden reaction norms’, (Ghalambor et al. 2007)]. The presence of such interactions between genotype and nutritional environment suggest that anthropogenic change in nutrient availability may sometimes act upon underlying genetic variation in nutrient-based life-history strategies, leading to rapid evolutionary changes in life histories and nutritional responses (Table1: H14). For example, artificial selection on growth rate has resulted in changes in nitrogen assimilation ability and nitrogen composition of modern wheat varieties (Chapin 1980; Acquisti et al. 2009): slow-growing historical wheat cultivars were more efficient in taking up and utilizing inorganic nitrogen from nitrogen-poor soils compared to fast-growing recent cultivars (Foulkes et al. 1998). Such evolutionary changes in the ability to acquire and assimilate nutrients can be rapid, especially for nutrient-related traits with a simple genetic basis such as root hair expression in *Arabidopsis* which affects performance in low phosphorus environments (Bates and Lynch 2001). In another example, artificial selection experiments have shown that fat storage in response to high carbohydrates can evolve within eight generations (Warbrick-Smith et al. 2006).

While some case studies may show clear signatures of changing nutrition on the evolution of nutrient use and life-history traits, it is important to note that nutrient use is a composite trait driven by, and interacting with, a multitude of loci and metabolic pathways, highlighting the potential for complex and rapid evolutionary consequences of alterations to nutrient supply. It is likely that the evolutionary consequences of changes in nutrients depend on the nutrient in question, with some nutrients (e.g. phosphorus) promoting rapid adaptations, while others are associated with more constrained responses over time (e.g. sulfur, see Gresham et al. 2008). Because some elements are tightly linked in biology (e.g. nitrogen and phosphorus), we might expect correlated evolution in physiological traits determining their use efficiencies (Werner et al. 1998). In addition, as once-limiting nutrients increase in availability, other key nutrients may become limiting, resulting in a change in selection intensity across components of nutrient use and life-history allocation (Table1: H4). For instance, recent work demonstrated that *Daphnia* resurrected from resting eggs in 700-year-old sediments were more efficient in phosphorus use (Frisch et al. 2014) and less efficient in carbon use (Chowdhury et al. 2015) compared to descendants alive today. In pre-eutrophication (low phosphorus) conditions, it is likely that selection for efficient phosphorus use was high because algae are phosphorus limited and have more carbon than *Daphnia* requires (Jeyasingh 2007). Drawing upon these historical patterns should provide important insights into how contemporary and impending changes in ecosystem nutrient dynamics may shape the evolution of organismal life histories. Understanding genotype-by-nutrition interactions is one of the exciting empirical frontiers in the evolution of life histories in the anthropocene: to what extent does variable nutrition maintain such genetic variation and how does such standing variation contribute to rapid evolutionary responses to novel nutritional environments?

## Future Directions

Observations from the ecological literature on nutrient dynamics, coupled with theory and data from the life-history literature, suggest that changing nutrient availability may have major effects on life-history trade-offs and evolution in human-impacted environments. However, synthesizing this literature reveals many unknowns, some of which we highlight here as exciting future directions. In many ways, anthropogenic change in nutrients offers new opportunities for testing classic life-history theory. For instance, many laboratory studies have addressed the importance of genetic variation in resource acquisition in driving positive relationships between suites of life-history traits (King et al. 2011a,b; Robinson and Beckerman 2013), but fewer studies have addressed environmental variation in resources which may create similar positive correlations across life-history traits (van Noordwijk and de Jong 1986). Here, we discuss several open questions regarding the impact of anthropogenic nutrient change on life-history evolution.

### Effects of Spatial Variation in Changing Nutrient Availability

For many nutrients, changes in availability are not homogeneous across the landscape. While atmospheric nitrogen deposition has increased nitrogen availability across much of the eastern United States, increased phosphorus availability is relatively restricted to streams, lakes, and riparian areas. This suggests that changing nutrient availability may differentially affect certain species. However, some species may span a range of environments and thus experience an *increase in the variation* in nutrient availability across their range. This increased nutrient variation could have several effects, for instance, increasing selection on plastic responses to nutrient variation (Schlichting and Pigliucci 1998; Table1: H6) or amplifying variation in life-history trade-offs across populations. Increased spatial variation in nutrient availability could also produce variation across subpopulations in access to nutrients that might obscure fitness differences between genotypes (Figs4 and 5; van Noordwijk and de Jong 1986; Reznick et al. 2000) and result in changes in genotype frequency driven solely by variable resources (Table1: H13). Increased spatial variation in resource availability may even result in selection on novel habitat choice behavior for microhabitats with increased availability of a once-limited nutrient. For instance, increases in sodium availability along roadsides due to road salt runoff have been shown to result in novel preferences for roadside ponds in moose (Laurian et al. 2008). The extent to which these dynamics will drive evolutionary change will depend in part on individual mobility and gene flow between divergent nutritional contexts. Thus, comparisons between highly mobile or connected animal populations and less mobile or more highly fragmented taxa should be extremely useful in understanding the role of spatial scale. Similarly, as we discuss above, the consistency and heterogeneity of the nutritional environment that populations experience should have important consequences. In this context, organismal traits such as diet breadth offer important empirical opportunities. For example, comparisons between generalists and specialists or species associated with agricultural crops versus nondomesticated plant species offer critical contrasts for understanding how anthropogenic nutrient inputs affect different animal groups.

### Impact of changing nutrient dynamics on individual-level nutrition

At the ecosystem level, it is clear that nutrient cycling is changing. Here, we have focused on nutrition at the elemental level because this is the language of ecosystem studies that have documented human-induced changes in nutrient cycling. However, it is not always clear how these changing elements and molecules translate into the nutrition experienced by individuals. Investigating and validating assumptions linking elemental change to nutritional change will be an important component of studies that investigate life-history evolution in the face of anthropogenic nutrient change. Increasing nitrogen availability tends to increase both plant biomass and foliar nitrogen (Hwang et al. 2008), but the degree of these impacts varies across species (Magill et al. 1997). Nitrogen addition also affects how plants invest in defensive chemicals, often leading to a decrease in defenses (Prudic et al. 2005), but this effect also varies across species (Hwang et al. 2008). These variable responses of plants to nitrogen availability will in turn have variable effects on the herbivores of these plants. At the same time, the nutritional health of an individual is a composite of many nutrients, and different elements may be changing in different ways (Sardans et al. 2012). In some cases, a change in one nutrient may result in an overall nutrient imbalance (Fenn et al. 1998), which can have important consequences on growth and development (Raubenheimer and Jones 2006), although some organisms can compensate for nutrient imbalances through changes in feeding behavior (Raubenheimer and Simpson 1993; Morehouse and Rutowski 2010a; Jensen et al. 2011). These examples illustrate variable effects of changing nutrients on plant nutrition with respect to herbivores, but of course there will also be cascading effects across trophic levels in nutritive content (Schumacher and Platner 2009). Overall, an exciting area of future research will be translating elemental approaches to anthropogenic resource change to other components of nutrition such as digestible protein or carbohydrates (Simpson and Raubenheimer 2011).

### Impact of changing nutrient availability on life-history evolution in the field

This review has outlined several important areas where changing nutrient availability may affect the expression of life-history trade-offs and subsequent life-history evolution, such as loss of ornament honesty within the context of female choice, or relaxation of trade-offs between life-history traits which may affect survival in novel environments. However, many of these highlighted implications are based on inferences from theory and laboratory studies. We need substantially more work conducted on these ideas in the field—are populations in areas of nutrient enrichment showing relaxation of life-history trade-offs? On the other hand, are populations in areas of decreasing nutrient availability, showing more pronounced trade-offs? Under what conditions are nutrients increasing to stressful levels? Are organisms shifting to use novel signals that are not affected by increasing nutrients that affect their honesty? To what extent may these changes affect population divergence in environments more or less affected by anthropogenic nutrient change? Answering these questions in a range of systems is key for predicting how populations will respond to novel and rapidly changing environments. Particularly promising systems are those where past genotypes can be resurrected either directly (e.g. *Daphnia*) or indirectly through ancient DNA sequencing, and systems which may be particularly affected by nutrient change such as those found in aquatic, agricultural, and urban/suburban areas. As a range of species are studied, it will also be important to address how nutrient change affects different trophic levels: are the effects of changing nutrient cycles dampened or amplified as one moves from producers to herbivores to carnivores? Addressing the role of nutrition in life-history evolution is especially important given that environmental change caused by humans is often greater in magnitude and pace than changes that organisms have experienced in the past.

### Integrating ecological and evolutionary responses to anthropogenic nutrient change

This review has focused on the evolutionary impacts nutrient change may exert on life histories. However, such evolutionary dynamics will no doubt interact with ecological responses to nutrient change, which have been extensively discussed in the ecological literature (Tilman et al. 1996; Tilman 1999; Suding et al. 2005). Changes in nutrients have immediate impacts on community composition (Krupa 2003; Tylianakis et al. 2008; Bobbink et al. 2010), often favoring weedy species. Thus, the evolutionary implications of nutrient change for life histories discussed here may apply most prominently to a subset of species favored in these conditions (Table1: H16)—these species may form the basis of future diversification events (Tilman and Lehman 2001). Considering the effects of changing nutrients on not only community composition, but also the evolution of life histories, which in turn may impact ecosystem processes, provides a new avenue of investigating eco-evolutionary dynamics (Fussmann et al. 2007; Schoener 2011) and linking these two fields of biology (Jeyasingh et al. 2014).

### Promising systems and routes for future study

Anthropogenic nutrient change presents an opportunity to test classic questions about life-history evolution while also investigating important predictions concerning organismal responses to rapid environmental change (Table1). But what are the most promising routes to addressing the long list of questions and hypotheses? We have briefly discussed several possible systems throughout this review, but here, we discuss more broadly how one might find a particularly promising system to address these questions.

To zero in on the effects of anthropogenic nutrient change, it is important to find a system that allows a contrast in exposure to changing nutrients between species, populations, or individuals. One approach is to contrast variation in anthropogenic nutrient inputs across space. This could be a geographic contrast, for instance, comparing populations within a species that span areas that differ in intensity of atmospheric nitrogen deposition (e.g. eastern versus western United States or along an elevation gradient in the Sierras) or a continental approach that contrasts patterns of diversification with worldwide variation in nutrient availability (Orians and Milewski 2007). One could also focus on a species that is facultatively associated with humans, agriculture, or other areas differentially affected by changing nutrient availability (e.g. lakes with different upstream nutrient usage). Another possibility is to focus within a geographic area, but contrast species that vary in diets differentially affected by anthropogenic nutrient change. For instance, effects of atmospheric nitrogen deposition and road salt runoff have differential effects on plant nutrition depending on plant species (Krupa 2003; Snell-Rood et al. 2014); studies could contrast herbivores that feed on plants more or less affected by changing nutrients.

For some systems, it may be possible to contrast the effects of changing nutrients over time. This may be particularly tractable for species that can be resurrected, such as *Daphnia* clones, dormant seeds, or microbial spores. If these species are amenable to laboratory culture, this may allow for particularly powerful, controlled experiments that test for effects of resource availability on acquisition and allocation and how such patterns have changed coincident with nutrient change. Another temporal approach would be making use of museum specimens, for instance, measuring change in life-history traits over time, or using ancient DNA techniques to look at evolutionary change aligned with shifting nutrient availability. Of course many of the hypotheses outlined here (Table1) could also be addressed through experimental evolution. Indeed, it may be of particular interest to contrast evolutionary responses to small-scale nutrient variation versus the types of changes seen with anthropogenic nutrient change—rapid, exponential changes in the mean and variance. Overall, a range of field and laboratory studies will be necessary to understand predictable patterns of life-history evolution in the anthropocene. We suggest that study of anthropogenic nutrient inputs may not only highlight the detrimental effects of these man-made ecosystem disturbances, they may also reveal important insights into longstanding questions regarding how organismal life histories evolve in conjunction with ecological variation.
